# Assessment of a digital and an analog PET/CT system for accurate myocardial perfusion imaging with a flow phantom

**DOI:** 10.1007/s12350-021-02631-9

**Published:** 2021-05-04

**Authors:** Reetta Siekkinen, Anna K. Kirjavainen, Kalle Koskensalo, Nadia A. S. Smith, Andrew Fenwick, Virva Saunavaara, Tuula Tolvanen, Hidehiro Iida, Antti Saraste, Mika Teräs, Jarmo Teuho

**Affiliations:** 1 Turku PET Centre, University of Turku and Turku University Hospital, Kiinamyllynkatu 4-8, Turku, 20521 Finland; 2 Department of Medical Physics, Turku University Hospital, Turku, Finland; 3 Department of Computing, University of Turku, Turku, Finland; 4 Radiopharmaceutical Chemistry Laboratory, Turku PET Centre, University of Turku, Turku, Finland; 5 National Physical Laboratory, Teddington, UK; 6 Heart Center, Turku University Hospital, Turku, Finland; 7 Department of Biomedicine, University of Turku, Turku, Finland

## Abstract

**Abstract:**

In Myocardial Perfusion Imaging (MPI) with Positron Emission Tomography/Computed Tomography (PET/CT) systems, accurate quantification is essential. We assessed flow quantification accuracy over various injected activities using a flow phantom.

**Methods:**

The study was performed on the digital 4-ring Discovery MI (DMI-20) and analog Discovery 690 (D690) PET/CT systems, using 325-1257 MBq of [^15^O]H_2_O. PET performance and flow quantification accuracy were assessed in terms of count-rates, dead-time factors (*DTF*), scatter fractions (*SF*), time-activity curves (TACs), areas-under-the-curves (AUCs) and flow values.

**Results:**

On DMI-20, prompts of 12.8 Mcps, *DTF* of 2.06 and *SF* of 46.1% were measured with 1257 MBq of activity. On the D690, prompts of 6.85 Mcps, *DTF* of 1.57 and *SF* of 32.5% were measured with 1230 MBq of activity. AUC values were linear over all activities. Mean wash-in flow error was − 9% for both systems whereas wash-out flow error was − 5% and − 6% for DMI-20 and D690. With the highest activity, wash-out flow error was − 12% and − 7% for the DMI-20 and D690.

**Conclusion:**

DMI-20 and D690 preserved accurate flow quantification over all injected activities, with maximum error of − 12%. In the future, flow quantification accuracy over the activities and count-rates evaluated in this study should be assessed.

**Supplementary Information:**

The online version of this article (10.1007/s12350-021-02631-9) contains supplementary material, which is available to authorized users.

## Introduction

Myocardial Perfusion Imaging (MPI) with dynamic Positron Emission Tomography (PET) allows quantitative measurements of absolute Myocardial Blood Flow (MBF).^1^ MPI assessed using PET and short-lived tracers is highly reproducible.^2^-^5^ However, physical performance of PET systems has been a key issue in achieving accurate flow quantification over a wide range of activities.^6^-^9^ PET systems are exposed to high count-rates and dead-time when activity is present in the injected vein and heart chamber, which may compromise image accuracy and quality.^10^ Due to dead-time effects, time-activity curves (TACs) from left ventricle blood pool and myocardial tissue may be underestimated, resulting in MBF quantification inaccuracy.^2^,^11^-^13^

With the introduction of digital PET/Computed Tomography (PET/CT) systems, improvements in count-rate performance and image quality have been shown with National Electrical Manufacturers Association (NEMA) tests.^14^,^15^ Therefore, there might be potential advantages in applying digital PET/CT systems in MPI quantification. Thus, the count-rate capabilities of digital systems need to be assessed with phantom studies at high count-rates representative to MPI measurements. Evaluation of flow quantification at various injected activities is of paramount importance to understand the effects of count-rate performance, which differs among PET systems.^6^,^7^,^9^,^16^

Most importantly, flow quantification accuracy of digital PET/CT systems over various count-rates should be evaluated not only on static phantoms but with phantoms simulating dynamic imaging conditions. In 16 the authors investigated perfusion quantification accuracy at various activities by using a PET/MR compatible flow phantom. A similar phantom modelling realistic cardiac flow was presented and validated for MPI in PET in Reference 17, allowing flow modelling and quantification against a ground truth value. Thus, PET system assessment using a flow phantom allows to evaluate both count-rate performance and flow quantification accuracy.

In this study, we aimed at assessing flow quantification accuracy of a novel digital PET/CT system in comparison to an analog PET/CT system. A state-of-the-art flow phantom was selected to evaluate the accuracy of flow quantification for various activities up to 1260 MBq of [^15^O]H_2_O at scan start time.

## Materials and Methods

### PET/CT Systems

The digital Discovery MI with four detector rings (DMI-20) 15 and analog Discovery 690 (D690) (GE Healthcare, US) 18 PET/CT systems were assessed. Performance characteristics are summarized in Supplemental Table 1.

**Table 1:** Activities at scan start time with flow meter readings. Qpump was 200 mL/min and Qcyl was 60% of Qpump

Measurement	Activity at scan start time		Qcyl		Qtube	
	MBq		mL/min		ML/min	
	DMI-20	D690	DMI-20	D690	DMI-20	D690
1	325	359	123	120	79.0	91.0
2	488	400	121	117	82.0	91.0
3	546	532	121	117	83.0	93.0
4	621	607	120	121	83.0	92.0
5	655	729	122	120	82.0	92.0
6	691	833	119	117	84.0	93.0
7	906	995	118	119	85.0	93.0
8	1060	1130	117	118	86.0	93.0
9	1257	1230	117	115	87.0	94.0
Mean ± SD	-	-	120 ± 2.2	118 ± 2.0	83.4 ± 2.4	92.4 ± 1.0

DMI-20 has 4 rings of silicon photomultiplier (SiPM) detectors. One detector block comprises 4 × 9 array of 3.95 × 5.3 × 25 mm LBS-crystals coupled to 3 × 6 array of SiPM with 2 × 3 pixels. Transaxial and axial field-of-views (FOV) are 70 cm and 20 cm.^15^

D690 has 24 detector rings with photomultiplier tube (PMT) blocks. One block comprises 9 × 6 array of 4.2 × 6.3 × 25 mm LBS-crystals coupled to a single square PMT with 4 anodes. Transaxial and axial FOVs are 70 cm and 15.7 cm.^18^

Both systems employ similar randoms, scatter and dead-time corrections in image reconstruction: randoms from singles,^19^ model-based scatter correction with extension to time-of-flight (TOF) 20,21 and dead-time measured from the detector pile-up losses.^22^,^23^

### Flow Phantom System

To investigate flow quantification accuracy, a flow phantom (DCE Dynamic Flow Phantom, Shelley Medical Imaging Technologies, Canada) was used. Phantom validation for MPI in PET is presented in Reference 17.

The phantom set-up contains water container, peristaltic pump, injection port, phantom shell, flow constrictor valves and flow meters. The phantom schematic is shown in Supplemental Figure. 1. The shell has similar dimensions as the NEMA image quality phantom (diameter of 31 cm). An input chamber (volume of 15.7 mL) and an exchange cylinder (volume of 161 mL) containing a perforated tube (volume of 35 mL) are located inside the shell. The input chamber models the left ventricle blood pool whereas the exchange cylinder and perforated tube model tracer exchange in myocardial tissue. These allow to measure input and tissue TACs for kinetic modelling.

Flow inside the system is controlled with the peristaltic pump $\left( {Qpump} \right)$. Flow inside the exchange cylinder ($Qcyl$) and perforated tube ($Qtube$) are controlled with flow constrictor valves and recorded with microturbine flow meters (Omega Engineering Inc., US).^24^ Ideally, $Qtube$ and $Qcyl$ should be equal to $Qpump$. Reference flow ($Qref$) values are related to $Qcyl$ and derived from flow meter calibration using a lookup table. Further information from the flow values is given in Supplemental Figure. 1.

#### Kinetic Modeling of Flow Values

The input and tissue TACs can be measured by a volume-of-interest (VOI) analysis, defined for the input chamber and exchange cylinder, namely $C_{inputVOI} \left( t \right)$ and $C_{cylVOI} \left( t \right)$. VOIs were selected as in Reference 5. The TACs are used for image-based flow ($Qin, Qout$) modelling based on a two-compartment kinetic (one-tissue compartment) model as implemented in the phantom flow quantification software (QuantifyDCE 1.1, Shelley Medical Imaging Technologies, Canada).^17^

The software models tracer concentration in the exchange cylinder as:

1 $$ C_{cyl} \left( t \right) = q_{in} e^{{ - q_{out} t}} *C_{tube} \left( t \right), $$

where $*$ represents discrete convolution operation, $C_{cyl} \left( t \right)$ the time-dependent tracer concentration in the exchange cylinder, $q_{in}$ the tracer wash-in rate to the exchange cylinder (min^−1^), and $q_{out}$ the tracer wash-out rate from the exchange cylinder (min^−1^). $C_{tube} \left( t \right)$ represents tracer concentration in the perforated tube, which can be estimated from the input chamber TAC by transport delay of $C_{tube} \left( t \right) =$ $C_{inputVOI} \left( {t - delay} \right),$ when $t > delay.$ Moreover, as $C_{cylVOI} \left( t \right)$ contains contributions from both $C_{cyl} \left( t \right)$ and $C_{tube} \left( t \right)$, a signal mixing correction function is implemented as:

2 $$ C_{CylVOI} \left( t \right) = \left( {1 - ISF} \right) \times C_{tube} \left( t \right) + ISF \times C_{cyl} \left( t \right) $$

Thus, complete kinetic model consists of following free parameters:$$ q_{in} , $$ $$ q_{out} $$, $$ ISF $$ and $$ delay $$. Model fitting is performed with a weighted (by frame length) least squares algorithm.

The modeled parameters $q_{in}$ and $q_{out}$ can be considered analogous to rate parameters $K_{1}$ and $k_{2}$ in kinetic modelling of blood-tissue exchange. Final image-derived flow values $Qin$ and $Qout$ can be computed based on $q_{in}$ and $q_{out}$, multiplied with the cylinder volume ($V_{cyl} = 161 {\text{mL}}$), representing flow from the perforated tube to the exchange cylinder ($Q_{in} = V_{cyl} \times q_{in}$) and flow out from the exchange cylinder ($Q_{out} = V_{cyl} \times q_{out}$). Ideally, these flow values should be equal to the reference flow value, i.e. $Qin = Qout = Qref$ (mL/min). 17

### PET and CT Data Acquisition

At the start of the measurement, the flow meters, peristaltic pump (MasterFlex L/S, 07522-20, US) and [^15^O]H_2_O dispenser (Hidex Oy, Finland) were calibrated. Flow meter calibration and flow rates were fixed for all studies. $Qpump$ was set to 200 mL/min, while $Qcyl$ was adjusted to 60% of $Qpump$, following previous protocols.^5^,^17^

A CT-based attenuation correction (CTAC) using a tube voltage of 120 kV and current of 67mAs was acquired. The flow phantom was imaged in the center of the PET FOV. Dynamic list-mode PET acquisition was started 50 s after the dose injection, with a duration of 4 min and 40 s, following the clinical protocol.^25^ Measurements were repeated 9 times with individual doses. Injected doses were measured automatically by the [^15^O]H_2_O dispenser (Hidex Oy, Finland), which is cross-calibrated to a Veenstra VDC-404 (Veenstra Instruments, Netherlands) dose calibrator. Activities at scan start times and flow rates are reported in Table 1.

### Data Reconstruction

List-mode data was binned into 24 time-frames of 14 × 5 s, 3 × 10 s, 3 × 20 s and 4 × 30 s. On both systems, reconstructions were performed using three-dimensional ordered-subset-expectation-maximization (3D-OSEM) algorithm using point-spread function modelling (PSF) and TOF (vendor name: VUE Point FX-S), with 5 mm Gaussian post-filter and 35 cm FOV. On DMI-20, 3 iterations, 16 subsets and matrix size of 192 × 192 were used. On D690, 2 iterations, 24 subsets and matrix size of 128 × 128 were used. All data corrections including decay, attenuation, scatter, randoms and dead-time were performed.

### Flow Quantification Accuracy

Count-rate performance was assessed for each measurement by extracting count-rate data from DICOM headers. Specifically, peak prompts (*P*), randoms (*R*), dead-time factors (*DTF*) and scatter fractions (*SF*) corresponding the peak prompts were extracted.

Scatter rates (*S*) and trues rates (*T*) were derived based on the extracted *P*, *R* and *SF*. The scatter rate was derived as:

3 $$ S = SF*\left( {P - R} \right) $$

and the trues rate was derived as:

4 $$ T = P - S - R $$

We report the peak count-rates (*P*, *T*, *R*, *S*), *DTF* and *SF* as function of activity at scan start time.

Flow quantification accuracy was assessed in terms of shape of the input and tissue TACs and area-under-the-curve (AUC) of the TACs. Linearity of AUCs was determined with regression analysis of the AUC values versus activities at scan start time. We report AUCs and goodness-of-fits (R^2^) of the regression slopes.

Finally, accuracy of the modeled flow values $Q_{in}$ and $Q_{out}$ with respect to the reference flow $Q_{ref}$ was calculated as relative error:

5 $$ \% Error = \frac{{flow\;value {-} Q_{ref} }}{{Q_{ref} }} \times 100 \% , $$

where $flow\;value$ represents either $Q_{in}$ or $Q_{out}$ for each measurement. We report the accuracy of flow values with activity at scan start time. All flow values are reported as mean ± standard deviation (SD).

## Results

**Figure 1:**
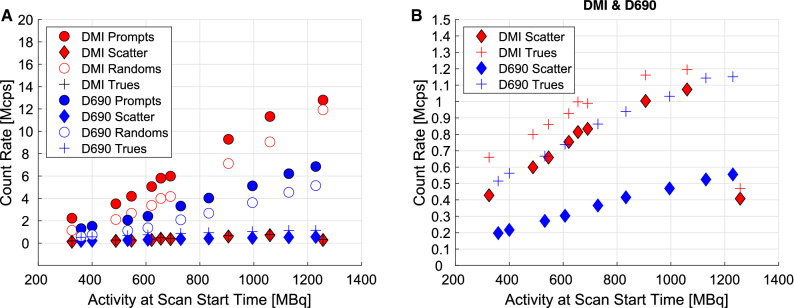
Count-rates for (a) prompts, scatter, randoms and trues and (b) zoomed-in plot for scatter and trues for DMI-20 and D690 systems for all flow phantom measurements as a function of activity at scan start time (Table 1)

Figure 1 shows peak prompts, scatter, randoms and trues as a function of activity at scan start time on DMI-20 and D690. On both systems, count-rates increased in relation to activities, as expected. For DMI-20, peak prompts were 12.8 Mcps with 1257 MBq. On D690, peak prompts were 6.85 Mcps with 1230 MBq. Additional data of the DMI prompts and randoms for the measurement with the highest injected activity (1257 MBq) is given in Supplementary Data 1.

**Figure 2:**
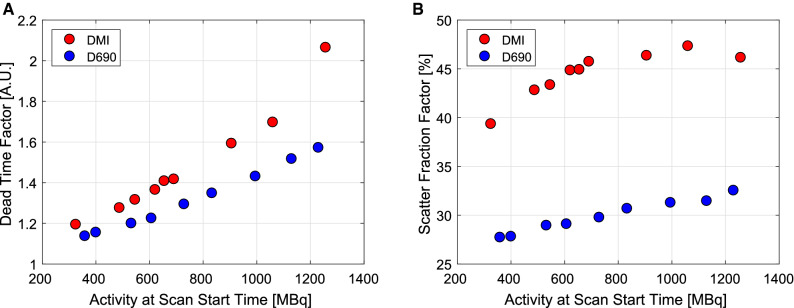
(a) Dead-time factors (*DTFs*) and (b) scatter fraction factors (*SFs*) at peak prompts as a function of activity at scan start time for all flow phantom measurements

*DTFs* as a function of activity at scan start time are presented in Figure 2A. *DTFs* increased nearly linearly in relation to activities, reflecting the count-rates (Figure 1). *DTFs* were higher with DMI-20 compared to D690. The highest *DTFs* 2.06 and 1.57 were measured with 1257 MBq and 1230 MBq on DMI-20 and D690.

Figure 2B shows *SF*s as a function of activity at scan start time. Due to larger axial FOV, *SFs* were higher on DMI-20 compared to D690. *SFs* increased up to the three highest activities (906 MBq, 1060 MBq, 1257 MBq at scan start time), after which *SFs* were nearly constant on DMI-20. On D690, *SFs* increased in relation to all activities.

**Figure 3:**
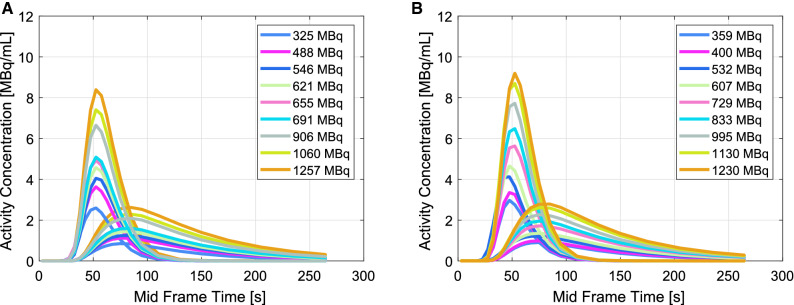
Time-activity curves (TACs) measured from the input chamber (input curve) and exchange cylinder (tissue curve) on (a) Discovery MI (DMI-20) and (b) Discovery 690 (D690) PET/CT system for all flow phantom measurements

Figure 3 shows TACs derived from the input chamber and exchange cylinder of the flow phantom. No visible distortions in TAC shapes were seen. Two-compartmental model fitting R^2^ values were 0.998 and 0.999 on both DMI-20 and D690.

**Figure 4:**
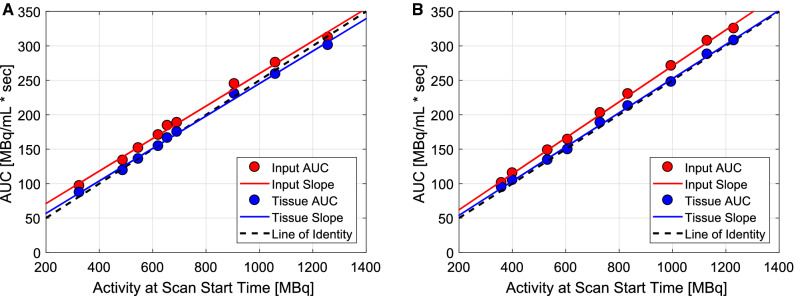
Areas-under-the-curves (AUCs) of the time-activity curves (TACs) on the (a) Discovery MI (DMI-20) and (b) Discovery 690 (D690) PET/CT system as a function of activity at scan start time with regression slope lines and line-of-identities for all flow phantom measurements

AUCs as a function of activity at scan start time with regression analysis are presented in Figure 4. R^2^ values for the input and tissue AUCs were 0.995 and 0.997 for DMI-20 and 0.998 and 0.998 for D690. AUCs increased linearly in relation to all activities on both systems. Tissue AUCs followed the line-of-identity closely whereas the input AUCs were above the identity line on both systems.

**Figure 5:**
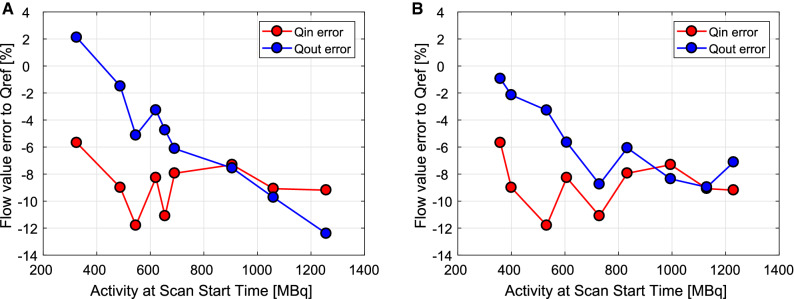
Errors of the modeled $Qin$ and $Qout$ flow values with respect to the reference flow $Qref$ measured on the (a) Discovery MI (DMI-20) and (b) Discovery 690 (D690) as a function of activity at scan start time for all flow phantom measurements

Prompts, *DTFs* and *SFs* with the accuracy of the modeled flow values are presented in Supplemental Table 2. Errors of the modeled flow values with respect to $Q_{ref }$ are presented as a function of activity at scan start time in Figure 5. A slight increase in $Q_{out}$ error was seen above activities of 906 MBq and 995 MBq at scan start time on DMI-20 and D690. In general, the error in flow quantification accuracy was small over all count-rates and activities. Also, the error magnitude of $Q_{in}$ and $Q_{out}$ can be considered small (below 5%) between DMI-20 and D690.

## Discussion

We assessed the accuracy of flow quantification on digital (DMI-20) and analog (D690) PET/CT systems over various injected activities with a flow phantom, for the first time.

DMI-20 showed improved performance with higher prompts compared to D690 over similar activities (Figure 1), in line with 9 and NEMA performance studies.^15^ DMI-20 limits the amount of data passed to the acquisition sorter after reaching a throttle limit, due to its sensitivity. The throttle is specific for different ring configurations on DMI systems, limiting the amount of data transmitted for histogramming. The number of prompts in the DICOM header will be also capped at the scanner-specific limit due to the throttle. The DMI-20 specific 12.8 Mcps limit was reached at activity of 1257 MBq. Therefore, a sudden notch in scatter and trues rate in Figure 1A and B is seen, similarly as in Reference 16.

However, during reconstruction, a scaling factor based on the prompts seen by the coincidence processor and prompts transmitted to histogramming will be applied to preserve quantification despite the throttle. Moreover, the actual RFS estimate will be slightly lower than the value for randoms in the DICOM header above throttle, as no count losses are taken into account for the randoms value saved in the DICOM header. The interested reader is referred to Supplementary Data 1 for more information.

Higher *DTFs* and *SFs* (Figure 2) were seen on DMI-20 over all activities. *SFs* increased in relation to activities, stabilizing at the highest activities (Figure 2B). This might be caused by biased scatter scaling at high activities by counts outside the body contour or by increased detector scatter, which is usually seen in NEMA testing.^15^ However, we detected no CTAC-PET misalignment that could affect scatter scaling. In general, no artifacts were seen resulting from scatter overcorrection on either system.

For flow quantification, TACs showed no visible artifacts (Figure 3). Similarly, the input and tissue AUCs showed high linearity (R^2^ close to 0.99) and followed the line-of-identity well (Figure 4). Saturation of TACs and non-linearity of AUCs over activities of 594 MBq of [^18^F]F^−^ 16 and saturation of input TAC with activities of 1006-1491 MBq of [^82^Rb] 13 have been shown previously. Saturation or non-linearity of AUCs were not seen on either system, contrary to 16 and.^13^ However, the input AUCs were slightly higher compared to tissue AUCs, which can contribute to the difference seen between $Q_{in}$ and $Q_{out}$ values (Figure 5), as we have previously discussed in.^5^

In line with the AUC linearity, errors in the modeled flow values were similar over all activities and systems (Figure 5). Mean $Q_{in}$ error was -9% and mean $Q_{out}$ error was -5% and -6% on DMI-20 and D690. The small difference between systems is contributed by different reconstruction parameters, which were selected based on clinical protocols on each system. $Q_{out}$ error increased when activity increased, up to − 12% on DMI-20 at the highest activity, and up to 7% on D690. Our finding is similar to 13 measured with [^82^Rb], although the maximum error in $Q_{out}$ (− 12%) was smaller compared to the reported error of 22%. Similarly to Reference 5, there was a systematic offset between in $Q_{in}$ vs $Q_{out}$ (Supplementary Table 1) and offset of 10 mL/min in Qcyl and Qtube (Table 1), which results in systematic negative bias irrespective of count-rate performance or the PET systems.

As a limitation, flow quantification was assessed only up to 1257 MBq at scan start time due to reaching the maximum limit of our [^15^O]H_2_O cyclotron production rate. Most importantly, image quantification was preserved up to highest activities. Therefore, we recommend limiting the injected activities to within the investigated range pending further investigation into values outside of this range on DMI-20 and D690. In clinical MPI studies, the injected doses for e.g. [^82^Rb] might exceed the limit of 1260 MBq, as both European and North American guidelines recommend injected doses from 1100 to 1500 MBq of [^82^Rb].^26^-^28^ At our institute, the injected activity is approximately 500 MBq, which decays to approximately 400 MBq at scan start time on both systems in clinical [^15^O]H_2_O MPI studies.

As an extension, other DMI systems should be evaluated. However, we expect that different DMI systems should behave similarly in terms of count-rate linearity despite the system-specific throttling limit. Additionally, we encourage future assessments with other perfusion tracers, such as [^82^Rb] or [^13^N] and an alternative MPI software estimation, as we only assessed [^15^O]H_2_O and the flow quantification software designed specifically for the phantom (QuantifyDCE).

Finally, our study indicates accurate MPI with [^15^O]H_2_O can be achieved if PET is acquired under the activities and count-rates specified above, as in References 6, 7, 9, and 16. The advantage of using a flow phantom is two-fold: the reference flow is always known, and CT-PET data can be collected free of any motion artefacts or misalignment. Thus, the phantom protocol used in this study could be applied for MPI harmonization studies for several PET/CT systems, according to their count-rate performance and reconstruction methods.

### New Knowledge Gained

Flow quantification accuracy in MPI with PET was preserved over various injected activities on DMI-20 and D690 PET/CT systems. Mean flow quantification error was -9% for wash-in flow and − 5% and − 6% for wash-out flow on DMI-20 and D690, indicating similar flow quantification errors on both systems. As flow quantification error was − 12% at 12.8 Mcps prompts on the DMI and − 7% at 6.85 Mcps prompts on the D690, we assume DMI-20 system might offer potential benefits in MPI in PET.

## Conclusions

Flow quantification accuracy was preserved over activities of 325-1257 MBq and 359-1230 MBq at scan start time on the digital 4-ring Discovery MI PET/CT system and analog Discovery 690 PET/CT system using [^15^O]H_2_O. The measurement with the highest prompts rate of 12.8 Mcps with corresponding *DTF* of 2.06 showed flow quantification error of − 12% on DMI-20. On D690 the highest prompts rate of 6.85 Mcps with corresponding *DTF* of 1.57 showed an error of − 7%. Generally, Discovery MI 4-ring system might offer potential benefits in MPI PET, although future studies should be conducted to assess the system performance at higher injected activities and prompt rates. We recommend performing MPI under 1257 MBq of activity at scan start time and a prompts rate of 12.8 Mcps on DMI-20.

## Supplementary Information

Below is the link to the electronic supplementary material.

Supplementary material 1 (DOCX 264 kb)

Supplementary material 2 (PPTX 7419 kb)

## Abbreviations

MPI: Myocardial perfusion imaging
PET: Positron emission tomography
MBF: Myocardial blood flow
TAC: Time-activity curve
PET/CT: Positron emission tomography/computed tomography
NEMA: National Electrical Manufacturers Association
DMI-20: Discovery MI with 4 detector rings
SiPM: Silicon photomultiplier
FOV: Field-of-view
D690: Discovery 690

## References

[1] Danad I, Uusitalo V, Kero T, Saraste A, Raijmakers PG, Lammertsma AA (2014). Quantitative assessment of myocardial perfusion in the detection of significant coronary artery disease: Cutoff values and diagnostic accuracy of quantitative [15O]H2O PET imaging. J Am Coll Cardiol.

[2] Kero T, Nordström J, Harms HJ, Sörensen J, Ahlström H, Lubberink M (2017). Quantitative myocardial blood flow imaging with integrated time-of-flight PET-MR. EJNMMI Phys.

[3] Kaufmann PA, Gnecchi-Ruscone T, Yap JT, Rimoldi O, Camici PG (1999). Assessment of the reproducibility of baseline and hyperemic myocardial blood flow measurements with 15O-labeled water and PET. J Nucl Med.

[4] El Fakhri G, Kardan A, Sitek A, Dorbala S, Abi-Hatem N, Lahoud Y (2009). Reproducibility and accuracy of quantitative myocardial blood flow assessment with 82Rb PET: Comparison with 13 N-ammonia PET. J Nucl Med.

[5] Siekkinen R, Teuho J, Smith NAS, Fenwick A, Kirjavainen AK, Koskensalo K (2020). Study of the effect of reconstruction parameters for myocardial perfusion imaging in PET with a novel flow phantom. Front Phys.

[6] Renaud JM, Yip K, Guimond J, Trottier M, Pibarot P, Turcotteet E (2017). Characterization of 3-dimensional PET systems for accurate quantification of myocardial blood flow. J Nucl Med.

[7] De Kemp RA, Klein R, Renaud J, Alghamdi A, Lortie M, DaSilva JN (2008). 3D List-mode Cardiac PET for simultaneous quantification of myocardial blood flow and ventricular function. IEEE Nucl Sci Symp Conf Rec.

[8] Nazir MS, Gould S-M, Milidonis X, Reyes E, Ismail TF, Nejiet R (2019). Simultaneous 13 N-Ammonia and gadolinium first-pass myocardial perfusion with quantitative hybrid PET-MR imaging: A phantom and clinical feasibility study. Eur J Hybrid Imaging.

[9] van Dijk JD, Jager PL, van Osch JAC, Khodaverdi M, van Dalen JA (2019). Comparison of maximal Rubidium-82 activities for myocardial blood flow quantification between digital and conventional PET systems. J Nucl Cardiol.

[10] Nordström J, Kero T, Harms HJ, Widström C, Flachskampf FA, Sörensen J (2017). Calculation of left ventricular volumes and ejection fraction from dynamic cardiac-gated 15O-water PET/CT: 5D-PET. EJNMMI Phys.

[11] Moody JB, Lee BC, Corbett JR, Ficaro EP, Murthy VL (2015). Precision and accuracy of clinical quantification of myocardial blood flow by dynamic PET: A technical perspective. J Nucl Cardiol.

[12] Juneau D, Erthal F, Ohira H, Mc Ardle B, Hessian R, deKemp RA (2016). Clinical PET myocardial perfusion imaging and flow quantification. Cardiol Clin.

[13] Lassen ML, Manabe O, Otaki Y, Eisenberg E, Huynh PT, Wang F (2020). 3D PET/CT 82Rb PET myocardial blood flow quantification: Comparison of half-dose and full-dose protocols. Eur J Nucl Med Mol Imaging.

[14] National Electrical Manufacturers Association. Performance Measurements of Positron Emission Tomographs. NEMA Standards publication NU2-2012; 2012.

[15] Hsu DFC, Ilan E, Peterson WT, Uribe J, Lubberink M, Levin CS (2017). Studies of a next-generation silicon-photomultiplier-based Time-of-Flight PET/CT system. J Nucl Med.

[16] O’Doherty J, Chalampalakis Z, Schleyer P, Nazir MS, Chiribiri A, Marsden PK (2017). The effect of high count rates on cardiac perfusion quantification in a simultaneous PET-MR system using a cardiac perfusion phantom. EJNMMI Phys.

[17] Gabrani-Juma H, Clarkin OJ, Pourmoghaddas A, Driscoll B, Wells RG, deKemp RA (2017). Validation of a multimodality flow phantom and its application for assessment of dynamic SPECT and PET technologies. IEEE Trans Med Imaging.

[18] Bettinardi V, Presotto L, Rapisarda E, Picchio M, Gianolli L, Gilardi MC (2011). Physical performance of the new hybrid PETCT discovery-690. Med Phys.

[19] Stearns CW, McDaniel DL, Kohlmyer SG, Arul PR, Geiser BP, Shanmugam V. Random coincidence estimation from single event rates on the Discovery ST PET/CT scanner. 2003 IEEE Nucl Sci Symp Conf Rec (IEEE Cat No03CH37515); 2004. 10.1109/NSSMIC.2003.1352545.

[20] Ollinger JM (1996). Model-based scatter correction for fully 3D PET. Phys Med Biol.

[21] Iatrou M, Manjeshwar RM, Stearns CW (2007). Comparison of two 3D implementations of TOF Scatter Estimation in 3D PET. IEEE Nucl Sci Symp Conf Rec.

[22] Lewellen TK, Kohlmyer SG, Miyaoka RS, Schubert S, Stearns CW (1994). Investigation of the count rate performance of the General Electric Advance positron emission tomograph. Nucl Sci Symp Med Imaging Conf Rec.

[23] Stearns CW. Estimating an acquisition-specific NEC curve for PET acquisitions. 2003 IEEE Nucl Sci Symp Conf Rec (IEEE Cat No03CH37515), vol. 4, p. 2578-80. 10.1109/nssmic.2003.1352417.

[24] Driscoll B, Keller H, Jaffray D, Coolens C (2013). Development of a dynamic quality assurance testing protocol for multisite clinical trial DCE-CT accreditation. Med Phys.

[25] Maaniitty T, Stenström I, Bax JJ, Uusitalo V, Ukkonen H, Kajander S (2017). Prognostic value of coronary CT angiography with selective PET perfusion imaging in coronary artery disease. JACC Cardiovasc Imaging.

[26] Murthy VL, Bateman TM, Beanlands RS, Berman DS, Borges-Neto S, Chareonthaitawee P (2018). Clinical quantification of myocardial blood flow using PET: Joint position paper of the SNMMI cardiovascular council and the ASNC. J Nucl Med.

[27] Dilsizian V, Bacharach SL, Beanlands RS, Bergmann SR, Delbeke D, Dorbala S (2016). ASNC imaging guidelines/SNMMI procedure standard for positron emission tomography (PET) nuclear cardiology procedures. J Nucl Cardiol.

[28] Hesse B, Lindhardt TB, Acampa W, Anagnostopoulos C, Ballinger J, Bax JJ (2008). EANM/ESC guidelines for radionuclide imaging of cardiac function. Eur J Nucl Med Mol Imaging.

