# Supplementary material for: Assessment of a digital and an analog PET/CT system for accurate myocardial perfusion imaging with a flow phantom
Source: J Nucl Cardiol. 2021 May 4;29(4):1964–72. doi: 10.1007/s12350-021-02631-9 (PMC9345842; doi:10.1007/s12350-021-02631-9)
Supplement: Supplementary file 1 — Supplementary material 1 (DOCX 264 kb) [file 12350_2021_2631_MOESM1_ESM.docx]

**
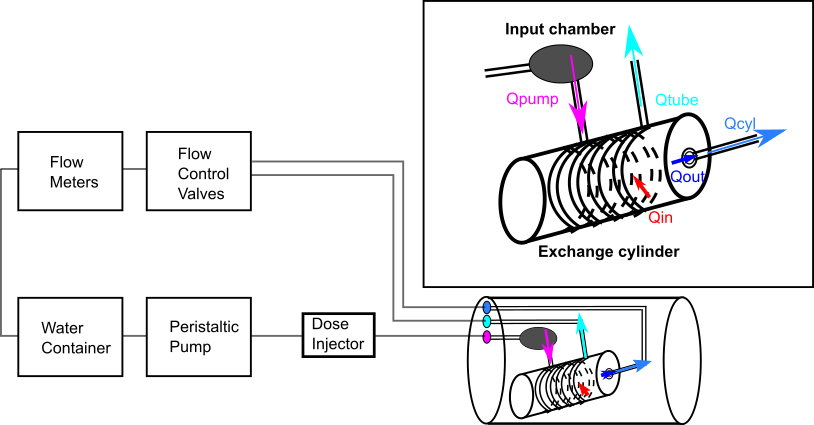
**

**Supplemental Fig. 1**  A schematic of the flow phantom set-up used in this study. The peristaltic pump runs water from the container to the closed-loop circulation with a fixed flow rate Qpump (mL/min). The radiotracer is given through the injection port. Water runs through the input chamber to the perforated tube inside the exchange cylinder. Flow inside the perforated tube (Qtube) and the exchange cylinder (Qcyl) are constricted with flow control valves and measured with flow meters. Modeled flow values Qin and Qout should be equal to Qcyl. (*1*)

**Supplemental Table 1.** Performance characteristics of the PET/CT systems according to the NEMA standard. (*2*,*3*)

|  |  | DMI-20 | D690 |
| --- | --- | --- | --- |
| Sensitivity | cps/kBq | 13.7 | 7.40 |
| Spatial Resolution | rad, 1 cm | 4.10 | 4.70 |
| Peak NECR | kcps | 193 | 139 |
| Peak NEC Activity | kBq/mL | 21.9 | 29.0 |
| Peak NEC Scatter Fraction | % | 40.6 | 37.0 |
| Timing Resolution | ps | 375 | 544 |
| Energy Resolution | % | 9.40 | 12.4 |

| **Measurement** | **Activity at Scan Start Time** | | **Peak Prompts** | | **DTF** | | **SF** | | **Qref** | | **Qin** | | **Qout** | | **Qin to Qref error** | | **Qout to Qref error** | | |
| --- | --- | --- | --- | --- | --- | --- | --- | --- | --- | --- | --- | --- | --- | --- | --- | --- | --- | --- | --- |
|  | MBq | | Mcps | | A.U. | | % | | mL/min | | mL/min | | mL/min | | % | | % | | |
|  | DMI-20 | D690 | DMI-20 | D690 | DMI-20 | D690 | DMI-20 | D690 | DMI-20 | D690 | DMI-20 | D690 | DMI-20 | D690 | DMI-20 | D690 | DMI-20 | D690 |  |
| 1 | 325 | 359 | 2.23 | 1.30 | 1.19 | 1.14 | 39.4 | 27.7 | 142 | 139 | 131 | 134 | 145 | 137 | -5.68 | -3.70 | 2.11 | -0.94 |  |
| 2 | 488 | 400 | 3.51 | 1.51 | 1.28 | 1.16 | 42.8 | 27.8 | 140 | 135 | 126 | 124 | 138 | 133 | -9.00 | -8.11 | -1.50 | -2.15 |  |
| 3 | 546 | 532 | 4.19 | 2.07 | 1.32 | 1.20 | 43.4 | 28.9 | 140 | 135 | 123 | 124 | 133 | 131 | -11.8 | -8.66 | -5.13 | -3.28 |  |
| 4 | 621 | 607 | 5.06 | 2.40 | 1.37 | 1.23 | 44.8 | 29.1 | 139 | 140 | 126 | 125 | 134 | 132 | -8.27 | -10.4 | -3.27 | -5.66 |  |
| 5 | 655 | 729 | 5.81 | 3.31 | 1.41 | 1.29 | 44.9 | 29.8 | 141 | 139 | 125 | 123 | 134 | 127 | -11.1 | -11.4 | -4.75 | -8.75 |  |
| 6 | 691 | 833 | 5.99 | 4.03 | 1.42 | 1.35 | 45.7 | 30.7 | 138 | 135 | 123 | 124 | 129 | 127 | -7.94 | -8.49 | -6.12 | -6.07 |  |
| 7 | 906 | 995 | 9.29 | 5.12 | 1.59 | 1.43 | 46.4 | 31.3 | 137 | 138 | 124 | 122 | 126 | 126 | -7.31 | -11.7 | -7.56 | -8.36 |  |
| 8 | 1060 | 1130 | 11.3 | 6.21 | 1.70 | 1.52 | 47.3 | 31.5 | 135 | 137 | 120 | 123 | 122 | 124 | -9.08 | -9.89 | -9.73 | -8.97 |  |
| 9 | 1257 | 1230 | 12.8 | 6.85 | 2.06 | 1.57 | 46.1 | 32.5 | 135 | 133 | 118 | 125 | 119 | 124 | -9.19 | -6.31 | -12.4 | -7.12 |  |
| Mean ± SD | - | - | - | - | - | - | - | - | 139 ± 2.4 | 137 ± 2.1 | 124 ± 3.8 | 125 ± 3.5 | 131 ± 8.0 | 129 ± 4.5 | -8.8 ± 1.9 | -8.7 ± 2.5 | -5.4 ± 4.3 | -5.7 ± 3.0 |  |

**Supplemental Table 2.** Accuracy of the modeled flow values with activities at scan start time and count-rates. Peak prompts, dead-time factors (DTF), scatter fractions (SF) and flow values ($Qref$, $Qin$, $Qout$) with error values (eq. 5 in manuscript).

**SUPPLEMENTAL DATA 1: VISUALISATION OF PROMPTS AND RANDOMS BELOW AND ABOVE THE THROTTLE RATE**

The scatter (S=SF*(P-R)) and trues (T=P-R-S) rates show a sudden notch with the measurement with the highest count-rate, compared to measurements with a lower injected activity (Figure 1A and 1B in the main manuscript). This is due to the system throttling, limiting the amount of prompts to 12.8 Mcps at high count-rates. How this affects the randoms from singles (RFS) estimate and how the system compensates for the throttle is shown in Supplemental Figures 2 and 3.

The rates presented in (Figure 1A and 1B) were extracted from the DICOM headers, which correspond to the randoms and prompts rates transmitted to histogramming. The randoms rates given by the RFS estimate and the amount of prompts seen by the coincidence processor are identical to what are seen in the DICOM header up to the throttle limit.

The randoms estimate based on the DICOM header data is higher above the throttle than the estimate calculated by RFS (Supplemental Figure 2). This is due to calculation of randoms from the RFS estimate including count losses whereas the randoms estimate from DICOM header does not take into account data losses. Image quantification is preserved after the throttle by implementing a scaling factor based on the ratio of prompts seen by the coincidence processor versus the amount of prompts transmitted to histogramming (Supplemental Figure 3).

For supplementary Figure 2, data from RFS estimate were extracted for comparison with the data contained in the DICOM header using the measurement with the highest activity (1257 MBq at scan start time) on DMI-20. For creation of Supplemental Figure 3, the prompts seen by the coincidence processor versus the data sent to histogrammer were plotted from the list-mode data using the measurement with the highest activity (1257 MBq at scan start time) on DMI-20.


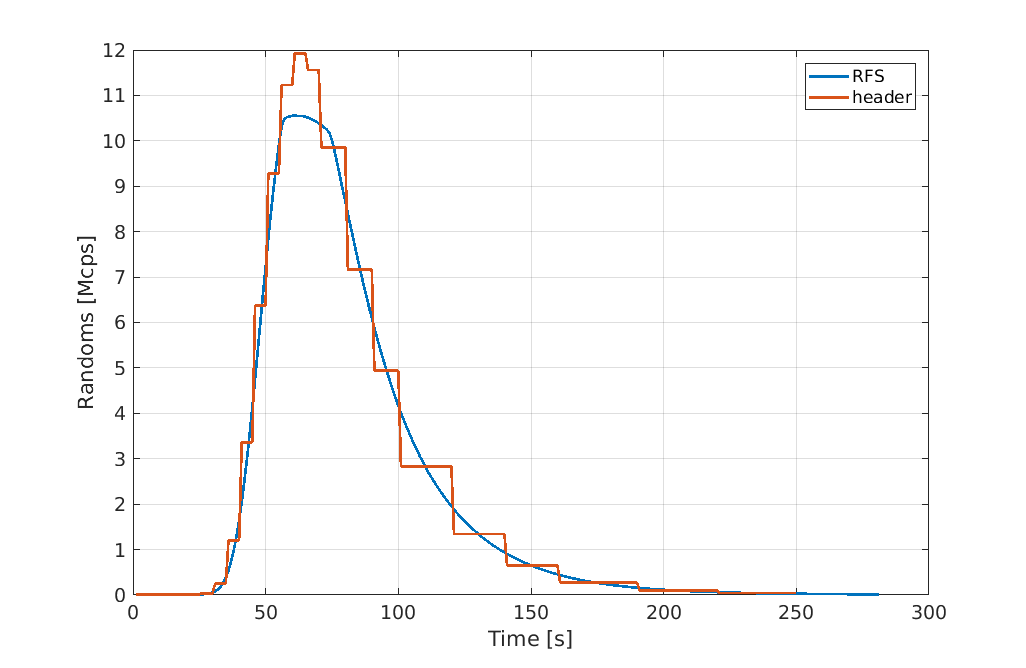


**Supplemental Fig. 2**  A presentation of randoms below and above the throttle rate of the DMI-20 system from the DICOM header (header) with randoms from randoms singles estimate (RFS). The rates are plotted across the duration of the measurement with the highest activity at scan start time (1257 MBq). Below throttle, randoms rates in the header and RFS are identical. Above throttle, RFS gives lower randoms rates compared to the header due to count-losses and live-time losses being taken into account. The count losses are mainly due to the increase of system dead-time. The header data is plotted per-frame basis as RFS is plotted per-second basis.


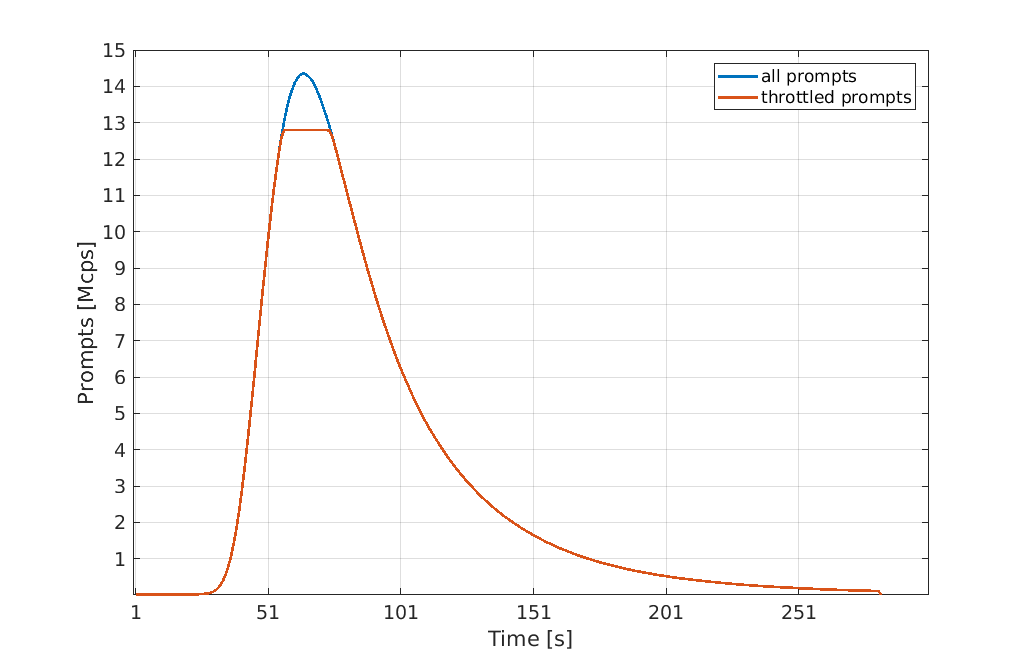


**Supplemental Fig. 3** A presentation of the prompts seen by the coincidence processor (14.3 Mcps) and the amount of prompts transmitted to histogramming before and after the throttling limit of the DMI-20 system (12.8 Mcps). The number of prompts transmitted to histogramming also equals the number saved in the DICOM header. After the throttling limit is reached, the relation of the detected (all) and transmitted (throttled) prompts is used to calculate a scaling factor inside the reconstruction, in order to ensure image quantification accuracy despite the throttle. The data is plotted as per-second data from the list-mode file.

**References**

1. Gabrani−Juma H, Clarkin OJ, Pourmoghaddas A, et al. Validation of a Multimodality Flow Phantom and Its Application for Assessment of Dynamic SPECT and PET Technologies. Vol 36.; :132−141.

2. Hsu DFC, Ilan E, Peterson WT, Uribe J, Lubberink M, Levin CS. Studies of a Next-Generation Silicon-Photomultiplier–Based Time-of-Flight PET/CT System. *J Nucl Med*. 2017;58:1511-1518.

3. Bettinardi V, Presotto L, Rapisarda E, Picchio M, Gianolli L, Gilardi MC. Physical Performance of the new hybrid PETCT Discovery-690. *Med Phys*. 2011;38:5394-5411.
