# Supplementary material for: Assessment of a digital and an analog PET/CT system for accurate myocardial perfusion imaging with a flow phantom
Source: J Nucl Cardiol. 2021 May 4;29(4):1964–72. doi: 10.1007/s12350-021-02631-9 (PMC9345842; doi:10.1007/s12350-021-02631-9)
Supplement: Supplementary file 2 — Supplementary material 2 (PPTX 7419 kb) [file 12350_2021_2631_MOESM2_ESM.pptx]

## Slide 1
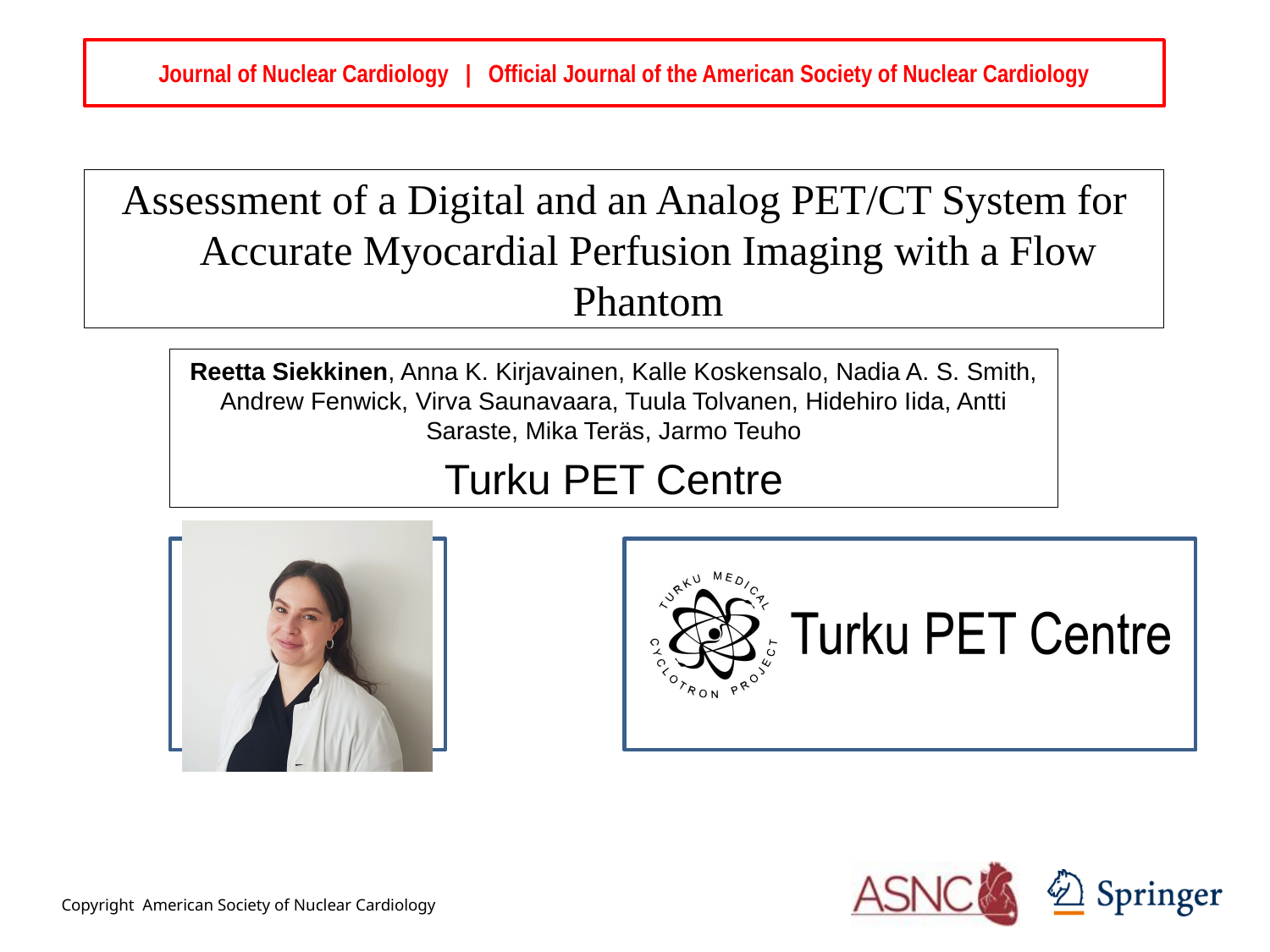

Journal of Nuclear Cardiology | Official Journal of the American Society of Nuclear Cardiology
# Assessment of a Digital and an Analog PET/CT System for Accurate Myocardial Perfusion Imaging with a Flow Phantom
Reetta Siekkinen, Anna K. Kirjavainen, Kalle Koskensalo, Nadia A. S. Smith, Andrew Fenwick, Virva Saunavaara, Tuula Tolvanen, Hidehiro Iida, Antti Saraste, Mika Teräs, Jarmo Teuho
Turku PET Centre
Head shot of author
required
Copyright American Society of Nuclear Cardiology

## Slide 2
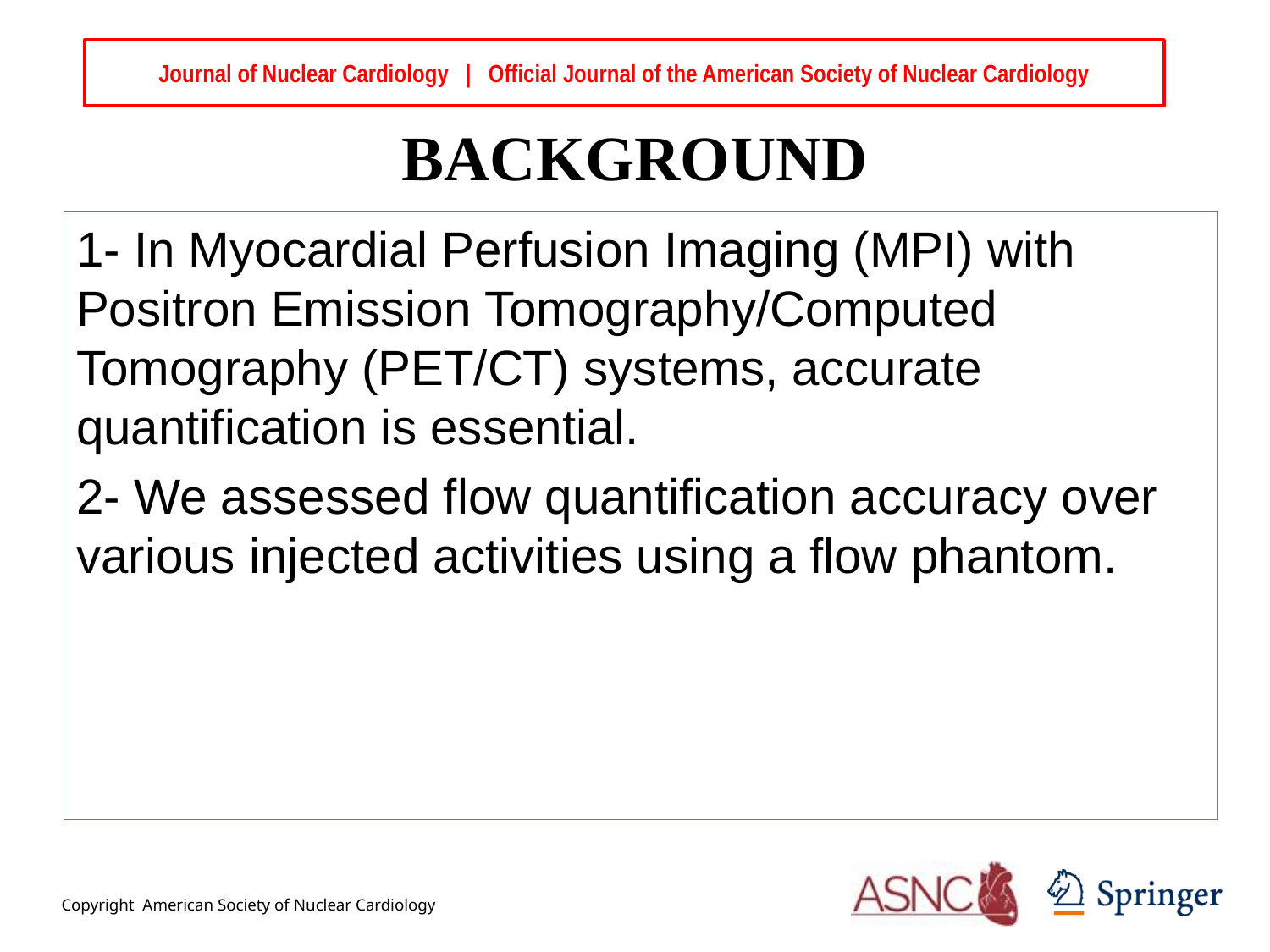

Journal of Nuclear Cardiology | Official Journal of the American Society of Nuclear Cardiology
# BACKGROUND
1- In Myocardial Perfusion Imaging (MPI) with Positron Emission Tomography/Computed Tomography (PET/CT) systems, accurate quantification is essential.
2- We assessed flow quantification accuracy over various injected activities using a flow phantom.
Copyright American Society of Nuclear Cardiology

## Slide 3
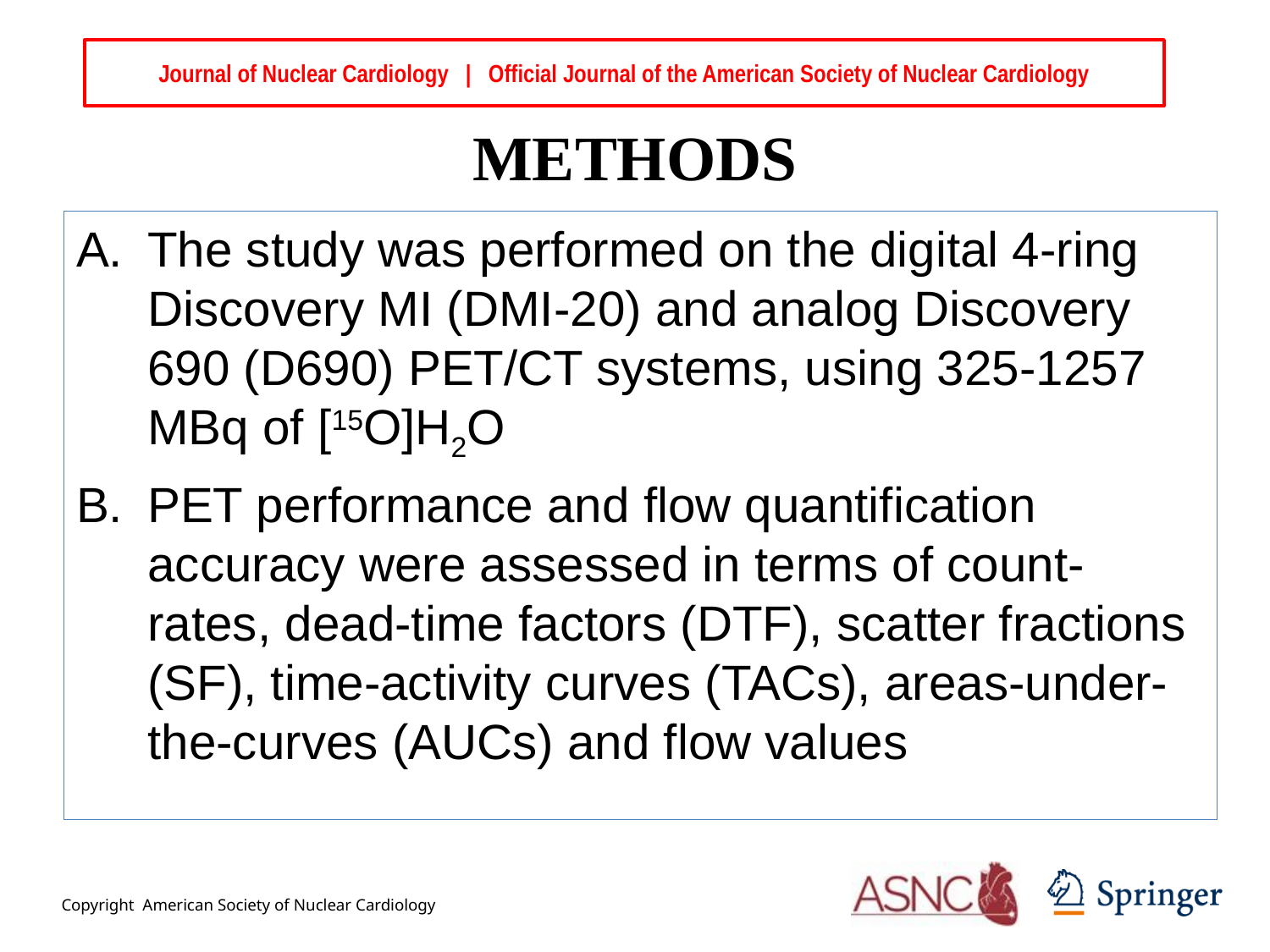

Journal of Nuclear Cardiology | Official Journal of the American Society of Nuclear Cardiology
# METHODS
The study was performed on the digital 4-ring Discovery MI (DMI-20) and analog Discovery 690 (D690) PET/CT systems, using 325-1257 MBq of [15O]H2O
PET performance and flow quantification accuracy were assessed in terms of count-rates, dead-time factors (DTF), scatter fractions (SF), time-activity curves (TACs), areas-under-the-curves (AUCs) and flow values
Copyright American Society of Nuclear Cardiology

## Slide 4
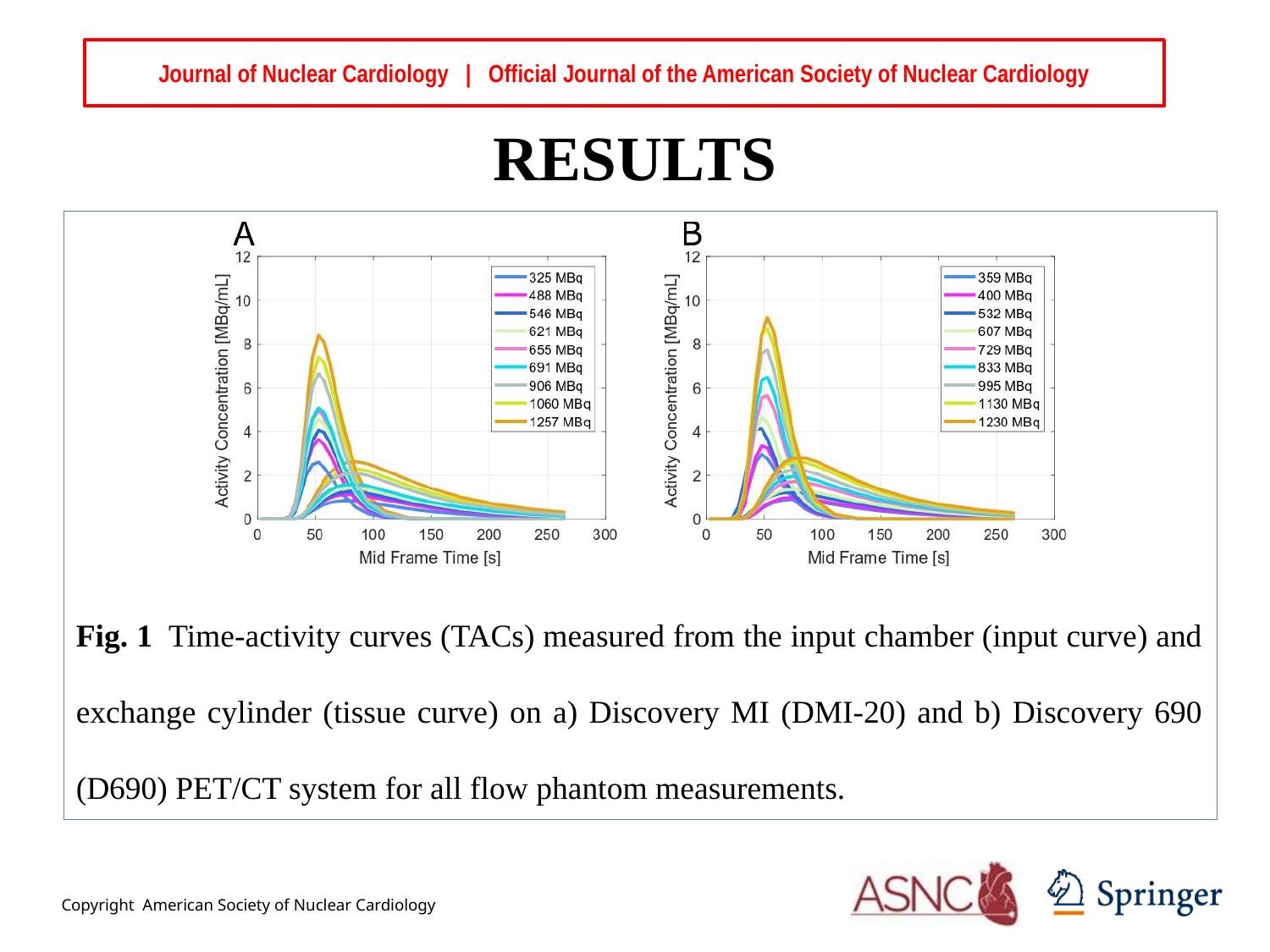

Journal of Nuclear Cardiology | Official Journal of the American Society of Nuclear Cardiology
# RESULTS
Fig. 1 Time-activity curves (TACs) measured from the input chamber (input curve) and exchange cylinder (tissue curve) on a) Discovery MI (DMI-20) and b) Discovery 690 (D690) PET/CT system for all flow phantom measurements.
Copyright American Society of Nuclear Cardiology

## Slide 5
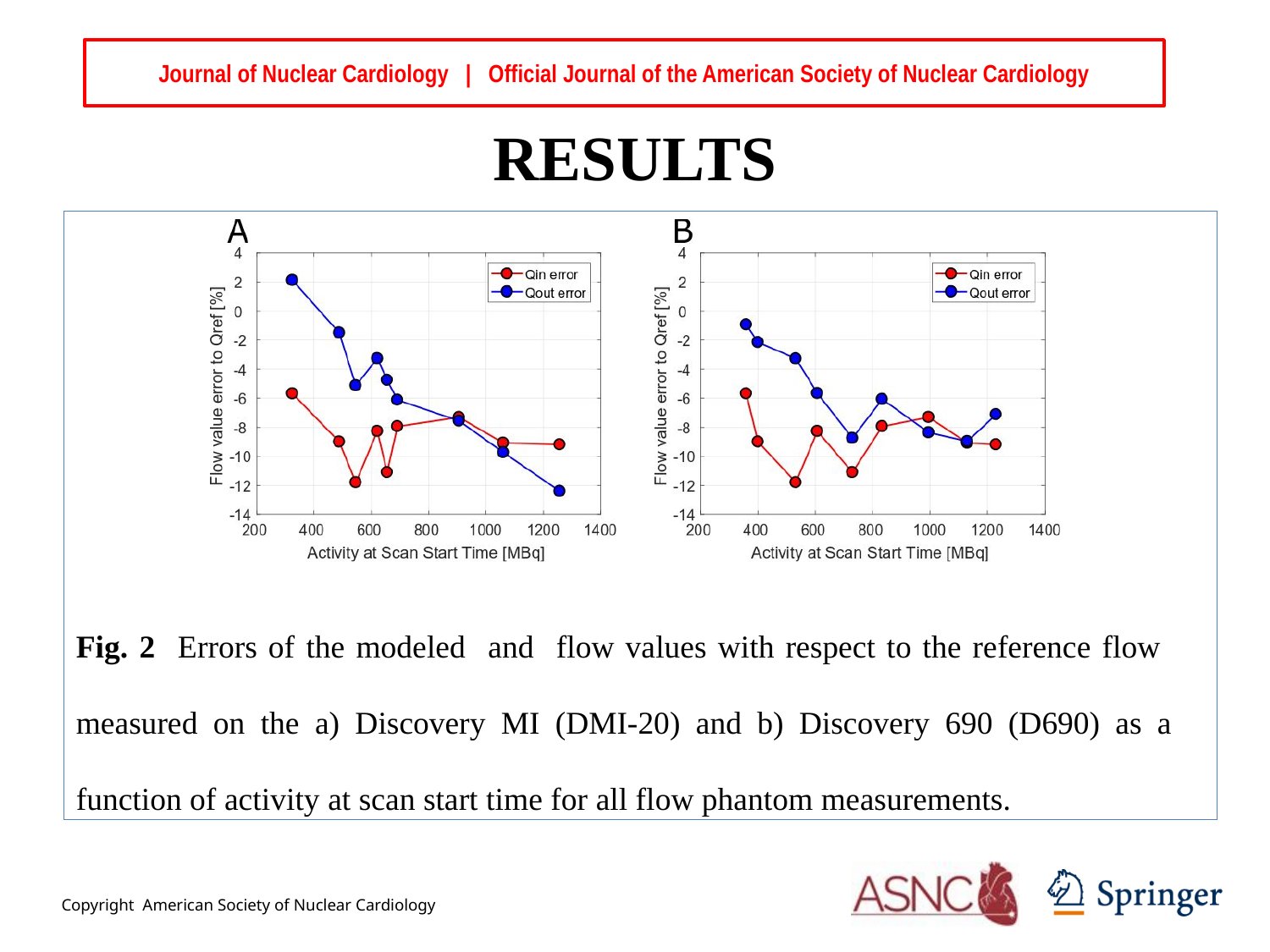

Journal of Nuclear Cardiology | Official Journal of the American Society of Nuclear Cardiology
# RESULTS
Insert a key table or a key figure
If figure, insert legend
Copyright American Society of Nuclear Cardiology

## Slide 6
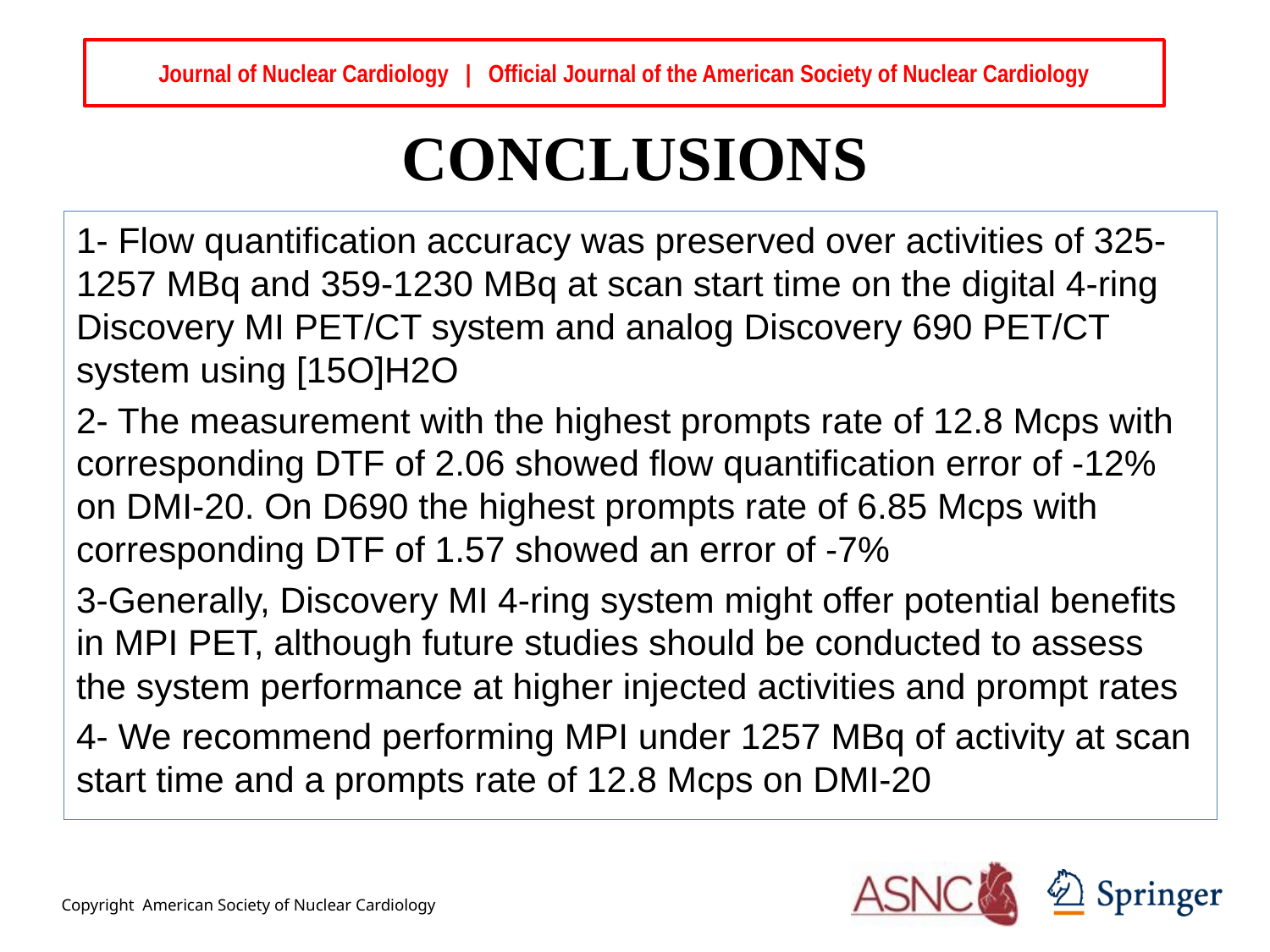

Journal of Nuclear Cardiology | Official Journal of the American Society of Nuclear Cardiology
# CONCLUSIONS
1- Flow quantification accuracy was preserved over activities of 325-1257 MBq and 359-1230 MBq at scan start time on the digital 4-ring Discovery MI PET/CT system and analog Discovery 690 PET/CT system using [15O]H2O
2- The measurement with the highest prompts rate of 12.8 Mcps with corresponding DTF of 2.06 showed flow quantification error of -12% on DMI-20. On D690 the highest prompts rate of 6.85 Mcps with corresponding DTF of 1.57 showed an error of -7%
3-Generally, Discovery MI 4-ring system might offer potential benefits in MPI PET, although future studies should be conducted to assess the system performance at higher injected activities and prompt rates
4- We recommend performing MPI under 1257 MBq of activity at scan start time and a prompts rate of 12.8 Mcps on DMI-20
Copyright American Society of Nuclear Cardiology
